# Pterostilbene suppresses oxidative stress and allergic airway inflammation through AMPK/Sirt1 and Nrf2/HO‐1 pathways

**DOI:** 10.1002/iid3.490

**Published:** 2021-08-02

**Authors:** Chang Xu, Yilan Song, Zhiguang Wang, Jingzhi Jiang, Yihua Piao, Li Li, Shan Jin, Liangchang Li, Lianhua Zhu, Guanghai Yan

**Affiliations:** ^1^ Jilin Key Laboratory for Immune and Targeting Research on Common Allergic Diseases Yanbian University Yanji Jilin China; ^2^ Department of Anatomy Histology and Embryology Yanbian University Medical College Yanji Jilin China; ^3^ Department of Respiratory Medicine Affiliated Hospital of Yanbian University Yanji Jilin China; ^4^ Department of Intensive Care Unit Affiliated Hospital of Yanbian University Yanji Jilin China; ^5^ Department of Dermatology Yanbian University Hospital Yanji Jilin China

**Keywords:** AMPK, HO‐1, Nrf2, oxidative stress, Pterostilbene (Pts), Sirt 1

## Abstract

**Introduction:**

Pterostilbene (Pts) may be used for allergic asthma treatment. The AMPK/Sirt1 and Nrf2/HO‐1 pathways are potential targets for asthma treatement. However, the relationship between Pts and AMPK/Sirt1 and Nrf2/HO‐1 pathways in asthma is unclear. Herein, we aim to explore the pharmacological effects of Pts on oxidative stress and allergic inflammatory response as well as the mechanism involving AMPK/Sirt1 and Nrf2/HO‐1 pathways.

**Methods:**

Asthma model was established in mice with ovalbumin (OVA). The model mice were treated by different concentrations of Pts. Lung pathological changes were observed through histological staining. In vitro, lipopolysaccharide (LPS)‐stimulated 16HBE cells were treated with Pts. The siAMPKα2, siSirt1 and siNrf2 knockdown, and treatment with compound C, EX‐527 or ML385 were also performed in 16HBE cells. Enzyme‐linked immunosorbent assay was used to detect interleukin‐4 (IL‐4), IL‐13, IL‐5, total and OVA specific immunoglobulin E (IgE), and interferon γ (IFN‐γ). Pneumonography was used to measure the airway hyperreactivity (AHR). Superoxide dismutase (SOD), catalase (CAT), and malondialdehyde (MDA) levels were also detected. Immunohistochemistry, Western blot and immunofluorescence were used to measure protein levels.

**Results:**

Pts significantly attenuated lung inflammatory cell infiltration and goblet cell proliferation. Meanwhile, Pts treatment could reduce IL‐4, IL‐13, IL‐5, and IgE (total and OVA specific) levels in the asthma model mice. However, IFN‐γ in bronchoalveolar lavage fluid was elevated. In addition, Pts reduced AHR. We also found that Pts treatment promoted serum SOD and CAT, and reduced MDA. In vitro results showed that Pts treatment promoted iNOS, TNF‐α, COX‐2, IL‐1β, and IL‐6 expressions in 16HBE cells, prolonged G0/G1 phase of the cell cycle, and resulted in a shortened G2M phase. Moreover, we found that Pts promoted the phosphorylation of AMPK in 16HBE, and meanwhile inhibited the increase of ROS induced by LPS. Additionally, Pts treatment inhibited p‐AMPK, Sirt1, Nrf2 and HO‐1, which in turn leads to the alleviation of AMPK/Sirt1 and Nrf2/HO‐1 pathways.

**Conclusion:**

Pts alleviated oxidative stress and allergic airway inflammation via regulation of AMPK/Sirt1and Nrf2/HO‐1 signaling pathways.

## Graphical abstract

Pts can relieve asthma by suppressing oxidative stress through the AMPK/Sirt1 and Nrf2/HO‐1 signaling pathways. Thus, Pts might be a potential anti‐inflammatory drug for the treatment of asthma.

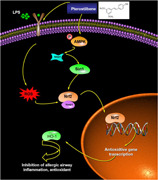

## 1 INTRODUCTION

Bronchial asthma is an allergic chronic airway inflammatory disease, and oxidative stress plays a key role.^1^ The oxygen free radicals produced by oxidative stress can cause damage to cells.^2^ Damaged cells can release active factors, including interleukin (IL)−6, IL‐1β, nitric oxide (NO), prostaglandin E2 (PGE2), and tumor necrosis factor‐α (TNF‐α).^3^, ^4^ Asthma is also closely related to type 1T helper cell (Th1)/Th2 imbalance. Generally, Th2 cytokines are upregulated in bronchial asthma, while Th1 cytokines are downregulated.^5^, ^6^ Th2 cytokines can induce immunoglobulin E (IgE) production and airway goblet cell metaplasia, and further can promote the infiltration of eosinophils in inflammatory sites.^7^ However, Th1 cytokines, such as interferon γ (IFN‐γ), can regulate cellular immune responses, and inhibit the development of asthma by antagonizing Th2‐mediated immune responses and reducing IgE synthesis.^8^, ^9^

The adenosine 5'‐monophosphate‐activated protein kinase (AMPK)/Sirtuin 1 (Sirt1) and nuclear factor‐E2‐related factor 2 (Nrf2)/Heme oxygenase‐1 (HO‐1) pathways relate closely with development of asthma. Asthma regulates metabolism and energy consumption by activating AMPK/SIRT1/PGC1 α signaling pathway.^10^ It has been proved that the mechanism of drug alleviating asthma is related to MAPK, nuclear factor‐κB, and Nrf2/HO‐1 signaling pathway.^11^ Sirt1 is continuously activated in asthma, and inhibition of the Sirt1 pathway alleviates ovalbumin (OVA)‐induced asthma.^12^ AMPK is an enzyme complex that regulates energy metabolism and has Sirt1‐dependent anti‐inflammatory activity.^13^ In addition, Nrf2/HO‐1 signals are activated by a variety of chemicals involved in the immune response.^14^ Therefore, the AMPK/Sirt1 and Nrf2/HO‐1 pathways may became potential targets for inflammatory diseases, including asthma.

Pterostilbene (Pts), isolated from Chinese herb Pterocarpus indicus Wild, has the effects of detumescence and hemostasis.^15^ It is previously used treating pulmonary heart disease and acute lung injury.^16^, ^17^ Pts can not only reduce the synthesis of collagen and the thickness of the reticular basement membrane, but also relieve the hyperplasia of the airway wall by inhibiting the thickening of the smooth muscle layer.^15^ It is shown that Pts significantly alleviated benzo[a]pyrene‐induced airway remodeling.^18^ In addition, Pts could inhibit FcεRI pathway via LKB1/AMPK pathway in allergic response.^19^ Interestingly, Pts reduces oxidative stress.^20^ Moreover, Pts can activate AMPK/Nrf2/HO‐1 pathway, thereby reducing cardiac inflammation and oxidative stress.^20^ These results indicate that Pts may be used for allergic asthma treatment.

Here, the protective effects and mechanisms of Pts on oxidative stress and airway inflammation in asthmatic mice were studied. The findings imply that Pts may potentially serve as an anti‐inflammatory drug for asthma.

## 2 MATERIALS AND METHODS

ARRIVE guideline checklist has been complied.

### 2.1 Animals

A total of 40 BALB/c mice (Yanbian University Medical Center, China) were fed with standard food and water. This study was approved by Institutional Animal Care and Use Committee of Yanbian University.

### 2.2 Animal treatment and grouping

The mice were divided into vehicle (Control) group, OVA group, 30 mg/kg Pts group (OVA + Pts30), 50 mg/kg Pts group (OVA + Pts50), with n = 8 per group. To establish asthma models, mice were sensitized with OVA (10 μg; Sigma‐Aldrich.) and aluminum hydroxide adjuvant (1 mg; Pierce) by intraperitoneal injection on Days 1, 7, and 14. The Control group was given 200 μl saline as control. After sensitization, mice were subjected to challenge with 1% OVA aerosol particles (3–5 μm) for 20 min on Days 21, 22, and 23. Mice in Control group were challenged with phosphate‐buffered saline (PBS) for 20 min. Pts (30 and 50 mg/kg body weight, MedChemExpress, China) was administered once a day by oral gavage to mice of OVA + Pts30 and OVA + Pts50 groups for 7 consecutive days. Mice were euthanized by cervical dislocation within 24 h of final OVA challenge.

### 2.3 Bronchoalveolar lavage fluid (BALF) collection and cell count

Mice were anesthetized with 0.75% sodium pentobarbital. Then, bronchoalveolar lavage fluid (BALF) was collected after lavaging with PBS (1 ml) and the liquid recovery rate should exceed 85%. Then, cell number in BALF was counted after centrifugation and Diff‐Quik staining (Baxter). The whole process was repeated three times.

### 2.4 Airway hyperresponsiveness (AHR) assessment

Airway hyperresponsiveness (AHR) assessment was measured after OVA challenge. Briefly, baseline values were recorded in barometric plethysmographic chamber. The Mch (aerosolized methacholine, from 2.5 to 50 mg/ml) of increasing concentrations was sprayed. After each nebulization, the averaged values were recorded. The pauses (Penh) represent bronchopulmonary resistances, that is, (expiratory time/relaxation time − 1) × (peak expiratory flow/peak inspiratory flow). For every concentration of Mch, Penh percentage increase from baseline was calculated, and Penh at baseline was shown as 100%.

### 2.5 Enzyme‐linked immunosorbent assay

For the detection of cytokines and antibodies in BALF, including IL‐4 (Cat#: BMS613), IL‐5 (Cat#: BMS610; Invitrogen), IL‐13 (Cat#: KMC2221; Invitrogen), IFN‐γ (Cat#: BMS606INST; Invitrogen) and total IgE, OVA‐specific IgE (Cat#: EMIGH; Invitrogen). The OD450 nm was detected (Biotek Instruments, Inc.).

### 2.6 Histology analysis

The lung tissues were embedded followed by cutting into 4 µm microsections. Hematoxylin‐eosin (H&E) staining was performed to analyze bronchus morphology. The inflammation scores were evaluated as previously described.^21^ In addition, periodic acid Schiff (PAS) staining assessed goblet cell proliferation and mucus production.

### 2.7 Determination of SOD, CAT, and MDA activity

Serum was separated by low‐speed centrifugation (3000 r/min) for 15 min. The SOD kit (OD550 nm; NJJCBIO), CAT kit (OD405 nm; NJJCBIO) and MDA kit (OD523 nm; NJJCBIO) was used respectively for the detections. Different OD values were measured by a micro multifunctional panel reader (Biotek instrument, Inc.). Then, the activity of SOD and CAT in each group was calculated according to the kit instructions.

### 2.8 Cell treatment

The 16HBE cells were treated with lipopolysaccharide (LPS) (1 mg/L, 00‐4976‐03, Invitrogen) at 37°C for 24 h, and then cultured with 15 or 30 μM Pts at 37°C for 24 h. Accordingly, Control group (without drug treatment), LPS group (LPS), 15 μM Pts (LPS + Pts15) group, and 30 μM Pts (LPS + Pts30) group were set up. For protein inhibition, 16HBE cells were first treated with Compound C (AMPK inhibitor, Cat: HY‐13418A; MCE), EX‐527 (Sirt1 inhibitor, Cat: HY‐15452; MCE), or ML385 (Nrf2 inhibitor, Cat: HY‐100523; MCE) for 2 h and then with Pts (30 mg/kg) for 1 h. For small interfering RNA transfection, 16HBE cells were transfected with siControl (#6568; Cell Signaling Technology), siAMPK (#6620; Cell Signaling Technology), siSirt1 (#12241; Cell Signaling Technology) and siNrf2 (#5285; Cell Signaling Technology) groups with Lipofectamine 2000 (#11668027; Invitrogen). After 6 h, fresh DMEM was added to continue the culture for 48 h.

### 2.9 MTT assay

The 16HBE cells were plated into a 96‐well plate (1 × 10^4^ cells). After treating with Pts (0, 1, 10, 50, and 100 μM), the cells were cultured for another 24 h. After that, 100 μl of MTT (1 g/L, Sigma‐Aldrich) were added, and cultured for 4 h. Finally, DMSO (100 μl) was added. OD540 nm was measured (Bio‐Rad Labs).

### 2.10 Detection of NO and inflammatory cytokines

NO levels were measured with Griess (Promega). The positive control group was treated with 10 μM l‐NIL (HY‐12116; MAC). Cytokines secreted by 16HBE cells were measured with ELISA kits (R&D systems). The positive control for PGE2 used 10 μM NS‐398 (HY‐13913; MAC).

### 2.11 Flow cytometry

After treatment, the cells were collected and immobilized at 4°C in 70% ethanol for 12 h. After rinsing and centrifugation, cells were stained with 500 μl of propidium iodide (Beyotime). After incubation in the dark at room temperature for 30 min, a flow cytometer detected cell cycle (BECKMAN COULTER; Cytoflex).

### 2.12 ROS level detection

The cells were incubated for 24 h with Pts 15 μM and 30 μM for 24 h. After adding LPS (1 mg/L) for further treatment for 1 h, the total reactive oxygen species (ROS) analysis kit (Cat#88‐5930‐74; Invitrogen) was used for detection. The ROS content was analyzed on Cytoflex (Beckman Coulter) and fluorescence microscope (Biotek Instruments).

### 2.13 Western blot

Total, cytoplasm and nucleus proteins were obtained. Proteins were subjected to separation and membrane transfer. After blocking, membrane labeling with primary and secondary antibodies was performed. Finally, the bands were developed with ECL (Beyotime), and scanned with Chemi‐Doc (BioRad). Primary antibodies included nitric oxide synthase (iNOS) (1:1000, #13120; Cell Signaling Technology), cyclooxygenase‐2 (COX‐2) (#12282,1:1000; Cell Signaling Technology), β‐actin (#4970, 1:1000; Cell Signaling Technology), AMPK (#4185, 1: 1000; Cell Signaling Technology), p‐AMPK (#2535, 1: 1000; Cell Signaling Technology), Sirt1 (#9475, 1: 1000; Cell Signaling Technology), HO‐1 (#82206, 1: 1000; Cell Signaling Technology), Nrf2 (#12721, 1: 1000; Cell Signaling Technology), and glyceraldehyde 3‐phosphate dehydrogenase (#2118, 1: 1000; Cell Signaling Technology), and PCNA (#13110, 1: 1000; Cell Signaling Technology). The horseradish peroxidase‐conjugated secondary antibody (Cat #5151; Cell Signaling Technology) was used.

### 2.14 Immunohistochemical staining

The lung sections were subjected to antigen retrieval. Then, primary antibodies of p‐AMPK (#2535), HO‐1 (#82206), Nrf2 (#12721) and Biotin‐labeled secondary antibody (#5151) were added for incubation. All antibodies were from Cell Signaling Technology. Then, the lung sections were incubated with diaminobenzidine followed by hematoxylin counterstain. Five randomly selected fields were photographed.

### 2.15 Immunofluorescence staining

For lung specimens, Tris‐EDTA buffer (pH 9.0) was first used for heat mediated antigen retrieval. After blocking, incubation with p‐AMPK antibody (Abcam) primary antibody at 4°C overnight, and subsequently with AlexaFluor 488 labeled goat antirabbit IgG (Guduo) at room temperature for 1 h was performed. 4′,6‐Diamidino‐2‐phenylindole (DAPI) (Roche) was used to label cell nuclei. The 16HBE cells were seeded in Lab‐Tek Chamber slides at 1 × 10^5^ cells/well and then treated as above described. The cells were incubated with p‐AMPK antibody at room temperature for 2 h (Abcam), and subsequently with a FITC‐conjugated secondary antibody (Abcam) at room temperature for 1 h. Subsequently, cells were stained with 300 μl of DAPI (Roche) for 30 min. Finally, a laser confocal microscope (Biotek Instruments, Inc.) was used to observe the immunofluorescence.

### 2.16 Statistical analysis

All data analysis was performed with SPSS Version 20.0 (SPSS Inc.). The data were shown as mean ± *SD*. Differences were compared with analysis of variance followed by TUKEY's post hoc test. *p* < .05 suggests significance.

## 3 RESULTS

### 3.1 Pts reduces airway inflammation in asthma

**Figure 1:**
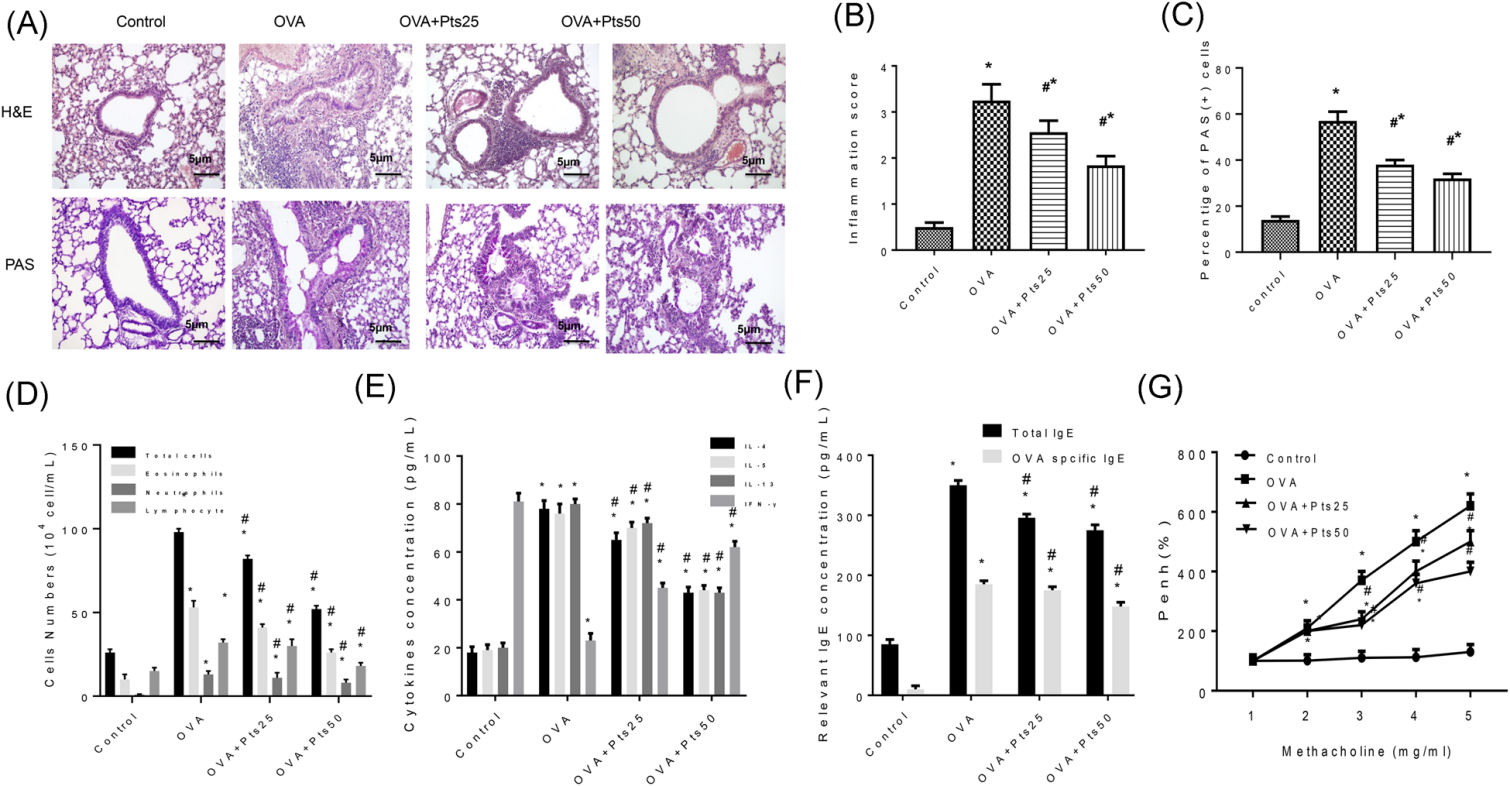
Pterostilbene reduces airway inflammation in OVA induced asthmatic mice. (A) The lung morphology was detected by H&E staining and the mucus around the airway was stained by PAS. Scale bar: 5 μm. (B) Inflammation score. (C) The percentage of positive PAS staining. (D) BALF cell count was determined after Diff‐Quik staining. (E) ELISA results of IL‐5, IL‐4, IFN‐γ, and IL‐13 in BALF. (F) ELISA results of IgE and OVA specific IgE in BALF. (G) AHR was measured with methacholine in OVA‐sensitized mice. All data were expressed as mean ± *SD*. *p < .05, compared with the Control group. ^#^ *p* < .05, compared with the OVA group. AHR, airway hyperresponsiveness; BALF, bronchoalveolar lavage fluid; ELISA, enzyme‐linked immunosorbent assay; H&E, haematoxylin and eosin; IFN‐γ, interferon‐γ; IgE, immunoglobulin E; IL, interleukin; OVA, ovalbumin; PAS, periodic acid Schiff

To determine whether Pts can alleviate allergic diseases, the lung morphology after treatment was observed after H&E staining. There were many infiltrating inflammatory cells in the OVA group around the airway (Figure 1A,B and *p* < .05). In contrast, the inflammatory cell infiltration in the OVA + Pts group was significantly reduced (Figure 1A,B and *p* < .05). Moreover, the PAS^+^ percentage was significantly reduced after Pts treatment, reflexing that Pts significantly reduces goblet cell production and mucus secretion dose‐dependently (Figures 1A,C and *p* < .05).

We further counted BALF cells after Diff‐Quik staining. Results showed that Pts dose‐dependently decreased the number of inflammatory cells (Figure 1D, *p* < .05). ELISA detection of inflammatory cytokines showed that Pts group had lower IL‐5, IL‐4, and IL‐13 levels, whereas higher IFN‐γ than OVA group (Figure 1E, *p* < .05). Similarly, total and OVA‐specific IgE in the Pts treatment group decreased significantly than OVA group (Figure 1F, *p* < .05). The Penh in conscious mice was used to detect AHR. Compared to Control, AHR in OVA group increased significantly, while Pts inhibited the increase of AHR (Figure 1G, *p* < .05). Thus, Pts can effectively suppress the recruitment of inflammatory cells, and reduce AHR, therefore attenuating the pathological response of asthmatic mice.

### 3.2 Pts attenuates inflammatory response in the LPS treated 16HBE cells

**Figure 2:**
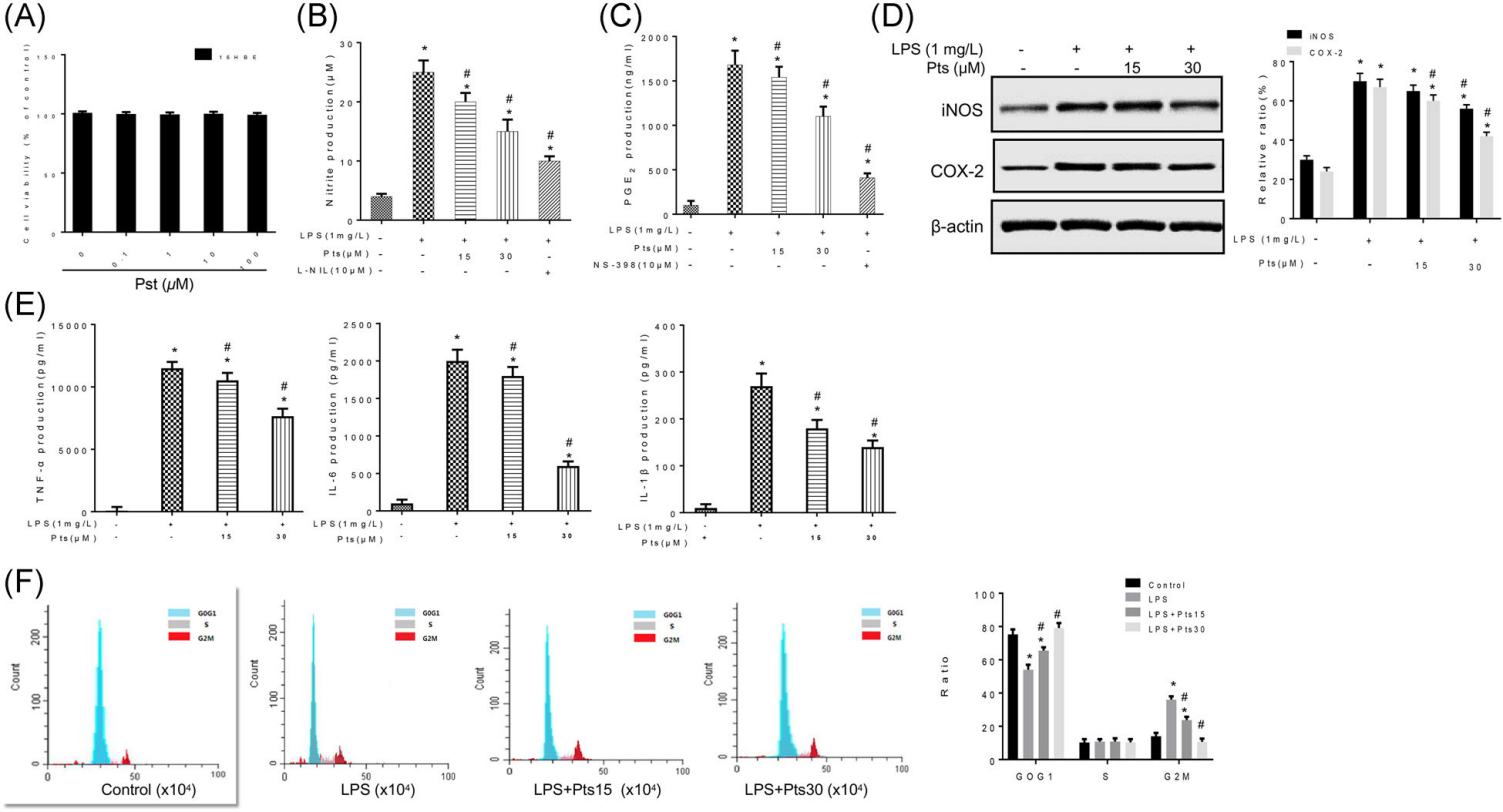
Pterostilbene attenuates inflammatory response in the LPS stimulated 16HBE cells. The 16HBE cells were treated with LPS with or without Pts (15 or 30 μM). (A) Cell viability was detected by MTT and represented by relative absorbance to control. (B) Determination of nitrite in 16HBE cells by the Griess reagent. (C) PGE2 production by 16HBE cells was measured with ELISA. (D) The iNOS and COX‐2 expressions were detected by Western blot. (E) Contents of IL‐6, TNF‐α, and IL‐1β was detected by ELISA. (F) Cell cycle of 16HBE cells was measured by flow cytometry. All data were expressed as mean ± *SD*. *p < .05, compared with the Control group. ^#^ *p* < .05, compared with the LPS group. ELISA, enzyme‐linked immunosorbent assay; IL, interleukin; LPS, lipopolysaccharide; MTT, 3‐(4,5‐dimethylthiazol‐2‐yl)‐2,5‐diphenyl‐2H‐tetrazolium bromide; Pts, Pterostilbene; TNF, tumor necrosis factor

MTT assay showed that cell viability of 16HBE was not apparently affected by Pts, even at a high concentration of 100 μM Pts (Figure 2A). Then the 16HBE cells were treated with LPS, followed by measurement of NO, PGE2, iNOS, and COX‐2, respectively. Resultantly, Pts dose‐dependently inhibited the levels of PGE2 and NO, as well as the levels of COX‐2 and iNOS (Figure 2B‐D, *p* < .05). ELISA detection of inflammatory cytokines showed that Pts dose‐dependently inhibited IL‐6, TNF‐α, and IL‐1β induced by LPS (Figure 2E, *p* < .05). To further study the effect of Pts on the cell cycle of 16HBE cells, flow cytometry was performed. LPS treatment prolonged the G0/G1 period and shortened the G2/M period. However, in the Pts treatment group, the G0/G1 phase was significantly shortened, and the G2/M phase was prolonged (Figure 2F, *p* < .05). Thus, Pts can down‐regulate the iNOS and COX‐2 and inhibit NO and PGE2. Moreover, Pts can down‐regulate IL‐6, TNF‐α, and IL‐1β.

### 3.3 Pts attenuates oxidative stress in vitro and in vivo

**Figure 3:**
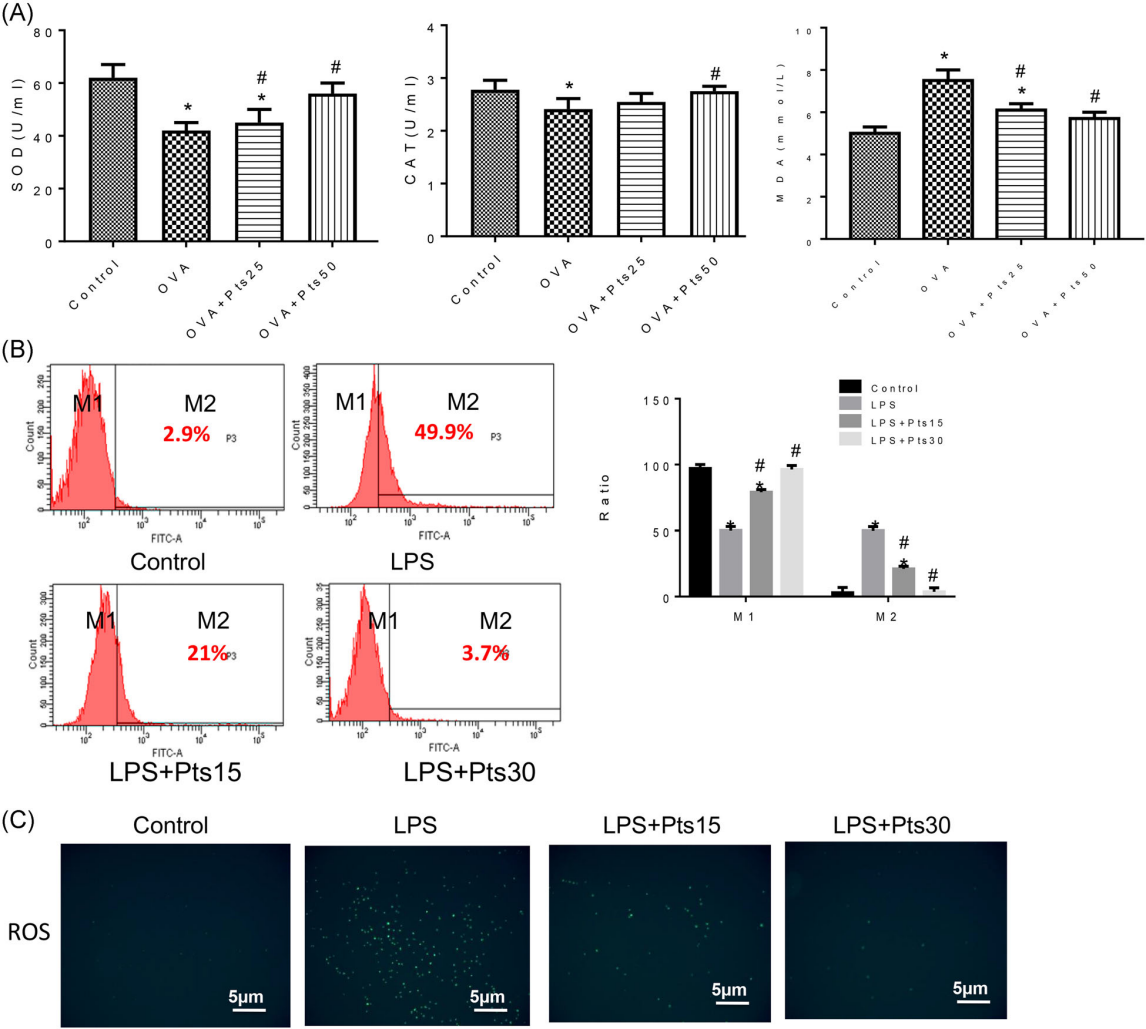
Pterostilbene attenuates oxidative stress in vitro and in vivo.(A) Serum SOD, CAT activity, and MDA level were measured by ELISA. (B) Flow technology method to detect ROS level. (C) Immunofluorescence method to detect ROS level. All data were expressed as mean ± *SD*. *p < .05, compared with the Control group. ^#^ *p* < .05, compared with the OVA group. CAT, catalase; ELISA. enzyme‐linked immunosorbent assay; LPS, lipopolysaccharide; MDA, malondialdehyde; ROS, reactive oxygen species; SOD, superoxide dismutase

We measured serum SOD, CAT and MDA levels to detect the effect of Pts on asthmatic oxidative stress. As in Figure 3A, PTS increased the levels of SOD and CAT levels in the serum of OVA group mice, whereas decreased MDA dose‐dependently. To measure the level of ROS after LPS stimulation in 16HBE cells, flow cytometry and immunofluorescence were performed. LPS treatment increased the ROS level of 16HBE, while Pts treatment could inhibit the expression of ROS (Figure 3B,C and *p* < .05). Therefore, Pts could enhance antioxidant capacity both in vivo and in vitro.

**Figure 4:**
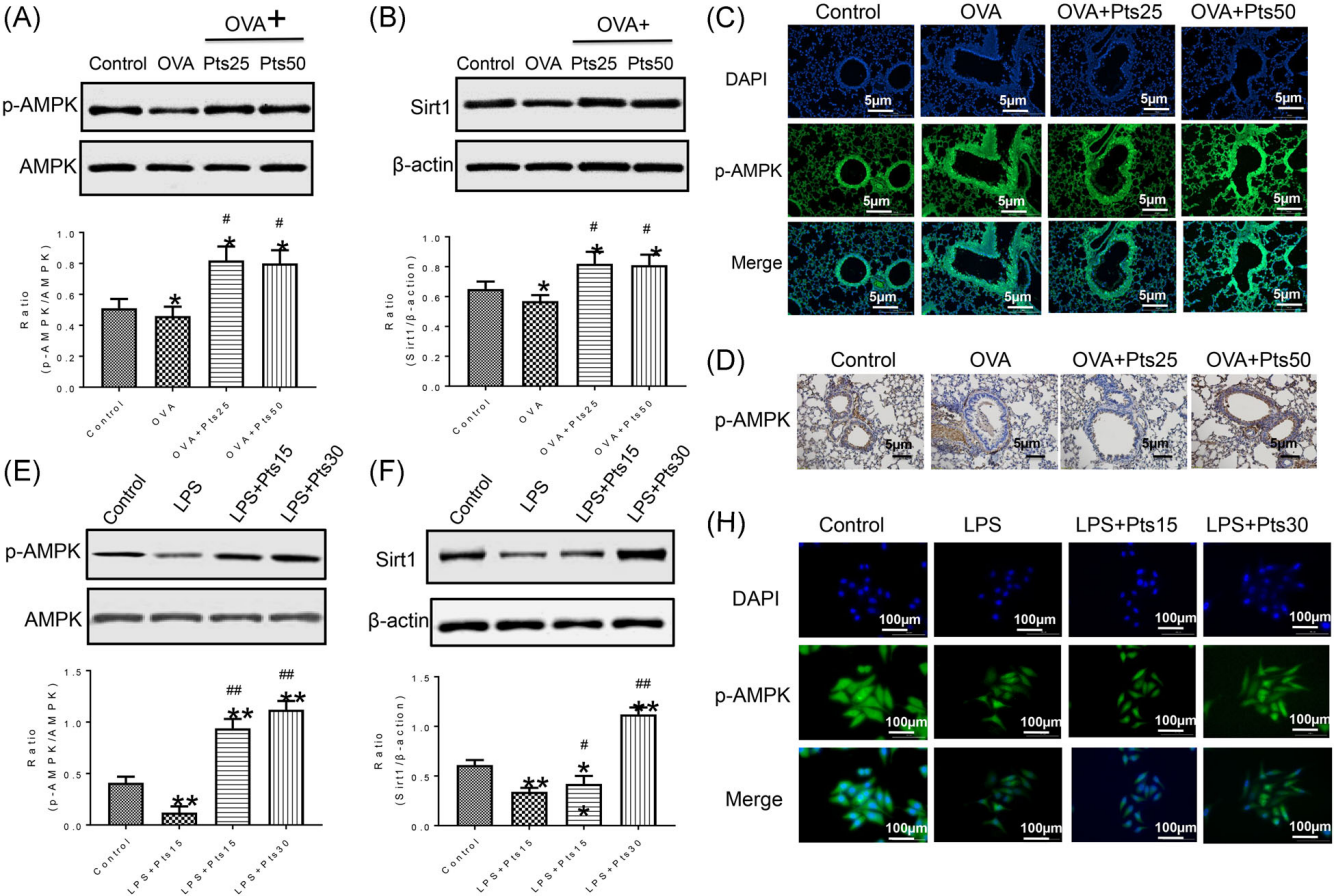
Pterostilbene activates AMPK/Sirt1 pathway. (A, B) Protein levels in lung tissues of mice were detected by western blot. (C) Detection of p‐AMPK protein expressioninmice by immunofluorescence. (D) P‐AMPK expression in mice was detected by immunohistochemical staining. (E, F) Protein levels in 16HBE cells were measured with Western blot. (H) The p‐AMPK in 16HBE cells was detected by immunofluorescence. All data were expressed as mean ± *SD*. *p < .05, **p < .01, compared with the Control group. ^#^ *p* < .05, ^##^ *p* < .01, compared with the OVA group or LPS group. AMPK, adenosine 5'‐monophosphate‐activated protein kinase; DAPI, 4′,6‐diamidino‐2‐phenylindole; LPS, lipopolysaccharide; OVA, ovalbumin

**Figure 5:**
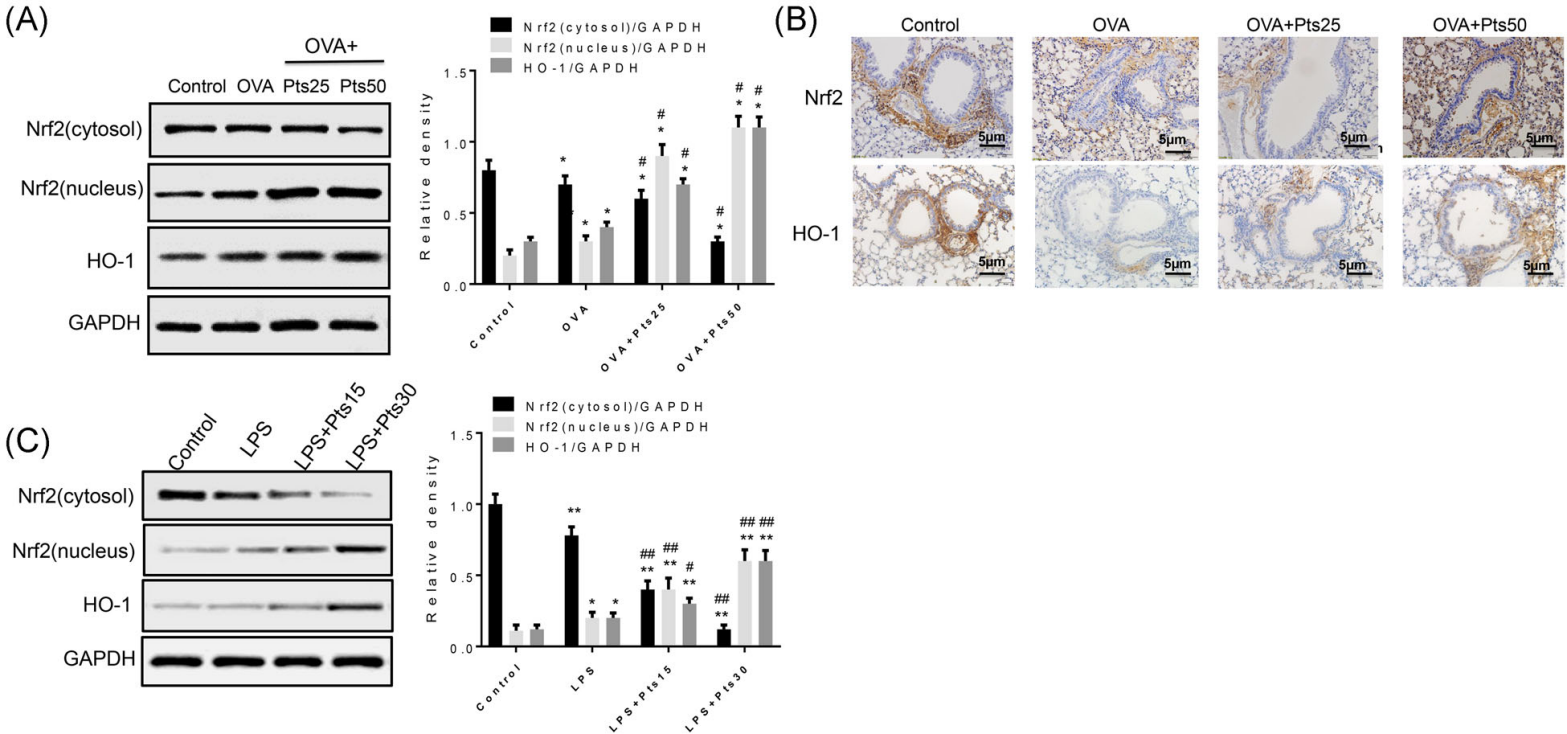
Pterostilbene activates Nrf2/HO‐1 pathway. (A) Protein levels in lung tissues were detected with Western blot. (B) Nrf2 and HO‐1 in lung tissues was detected by immunohistochemical staining. (C) Protein levels in 16HBE cells were detected with western blot. Scale bar = 5 μm. All data were expressed as mean ± *SD*. in vivo *p < .05, **p < .01, compared with the Control group. ^#^ *p* < .05, ^##^ *p* < .01, compared with the OVA group or LPS group. HO‐1, Heme oxygenase‐1; LPS, lipopolysaccharide; Nrf2, Nrf2, nuclear factor‐E2‐related factor 2; OVA, ovalbumin

### 3.4 Pts activates AMPK/Sirt1 pathway

We also detected p‐AMPK and Sirt1 proteins in lung and 16HBE cells by Western blot. Under the activation of inflammation, Pts promoted p‐AMPK and Sirt1 expressions (Figure 4A,B, *p* < .05, and Figure 4E,F, *p* < .01). Furthermore, immunofluorescence and immunohistochemistry were used to detect the changes of p‐AMPK in lung slices and in 16HBE cells (Figure 4C,D, and H), and the results were similar to that of Western blot analysis. Thus, we can speculate that Pts activates the AMPK/Sirt1 pathway.

### 3.5 Pts activates Nrf2/HO‐1 pathway

To further investigate the effect of Pts on Nrf2/HO‐1 pathway, we detected Nrf2 and HO‐1 expression with Western blot (Figure 5A). The model group had markedly higher Nrf2 (nucleus) and HO‐1 levels than control group. However, Pts increased their levels significantly (*p* < .05). And the results were validated by immunohistochemistry and Western blot analysis. Furthermore, Nrf2 and HO‐1 protein levels in 16HBE cells were consistent with that in lung tissues (Figure 5C, *p* < .01). Thus, Nrf2/HO‐1 pathway may be activated by Pts in vivo and in vitro.

### 3.6 Potential interaction between signaling pathways of AMPK/Sirt1 and Nrf2/HO‐1

**Figure 6:**
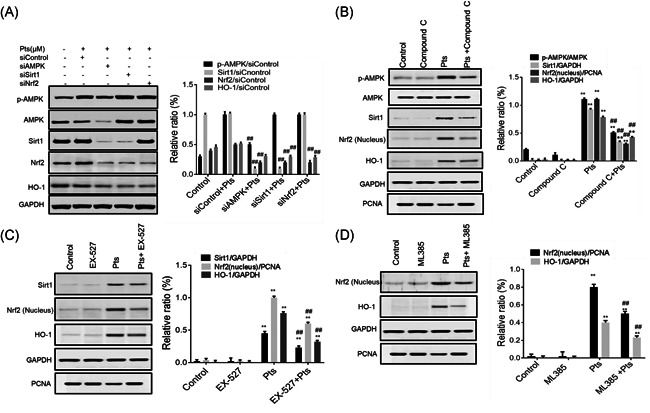
Potential interaction between signaling pathways of AMPK/Sirt1 and Nrf2/HO‐1. The 16HBE cells were treated as described in the method. The protein level was detected by western blot. (A) The levels of p‐AMPK, Sirt1, Nrf2 and HO‐1. ^##^ *p* < .01, compared with the siControl + Pts group. (B) p‐AMPK, Sirt1, Nrf2 and HO‐1 levels. **p < .01, compared with the Control group. ^##^ *p* < .01, compared with the Pts group. (C) Sirt1, Nrf2 and HO‐1 levels. **p < .01, compared with Control group. ^##^ *p* < .01, compared with the Pts group. (D) Nrf2 and HO‐1 levels. **p < .01, compared with Control group. ^##^ *p* < .01, compared with the Pts group. Each data value represents mean ± *SD*. AMPK, adenosine 5ʹ‐monophosphate‐activated protein kinase; GAPDH, glyceraldehyde 3‐phosphate dehydrogenase; HO‐1, Heme oxygenase‐1; Nrf2, nuclear factor‐E2‐related factor 2; Pts, Pterostilbene

Finally, to explore the association between AMPK/Sirt1 and Nrf2/HO‐1 pathways, we conducted knockdown experiments. Figure 6A showed that siAMPK treatment inhibited Sirt1 and Nrf2/HO‐1; siSirt1 reduced Nrf2/HO‐1 expression, and siNrf2 reduced HO‐1 expression (*p* < .01). To investigate whether Pts can regulate the AMPK/Sirt1 and Nrf2/HO‐1 pathways, we further pretreated 16HBE cells with compound C (AMPK inhibitor), EX‐527 (Sirt1 inhibitor) or ML385 (Nrf2 inhibitor). The results showed that compound C inhibited Sirt1, Nrf2, and HO‐1; EX‐527 inhibited Nrf2 and HO‐1; and ML385 could inhibit HO‐1 expression (Figures 6B,D, *p* < .01). In addition, in 16HBE cells, Pts activated p‐AMPK, Sirt1, Nrf2, and HO‐1. Pts + compound C treatment effectively blocked the activation of p‐AMPK, Sirt1, Nrf2 and HO‐1 in 16HBE cells, while Pts + EX‐527 also effectively inhibited Sirt1, Nrf2, and HO‐1. Pts + ML385 also effectively inhibited Nrf2 and HO‐1. Above all, Pts could activate signaling pathways of AMPK/Sirt1 and Nrf2/HO‐1 in LPS‐sensitized 16HBE cells.

**Figure 7:**
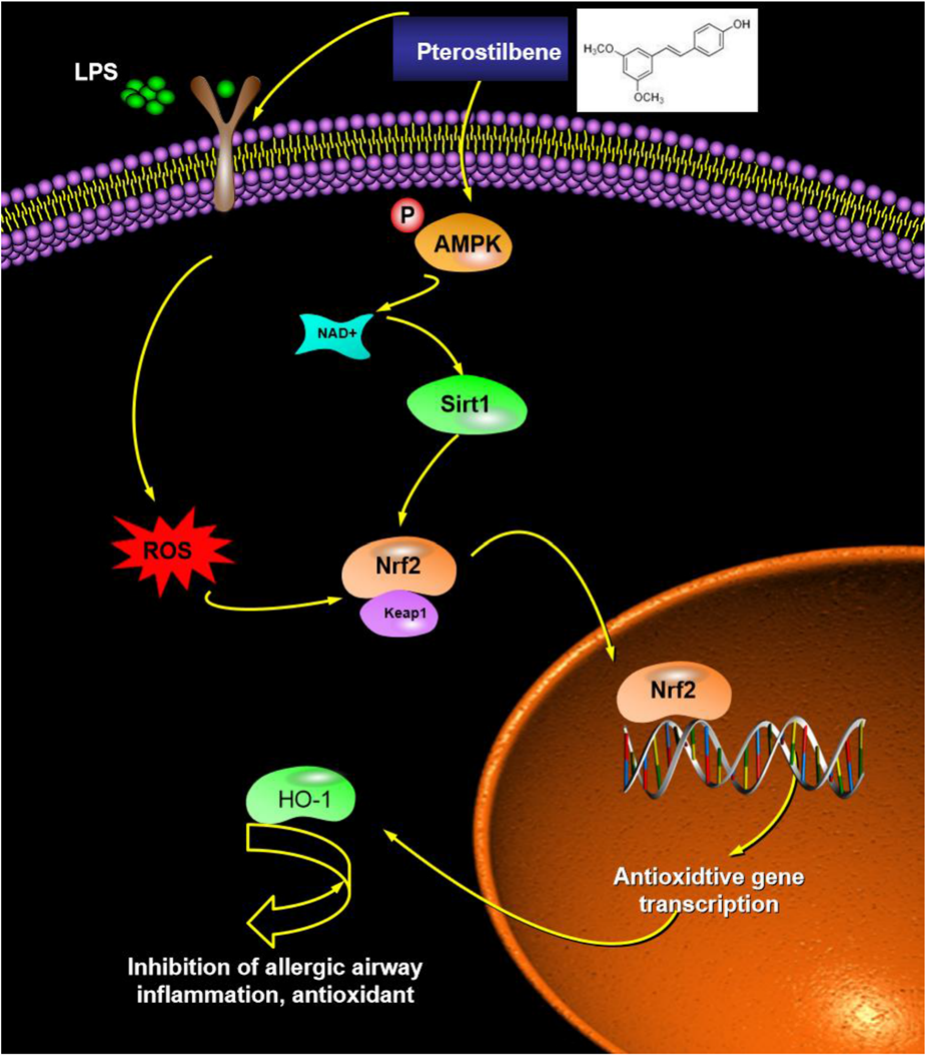
Schematic figure. Pts suppresses oxidative stress and allergic airway inflammation through AMPK/Sirt1/Nrf2/HO‐1 pathways. AMPK, adenosine 5ʹ‐monophosphate‐activated protein kinase; HO‐1, Heme oxygenase‐1; Nrf2, nuclear factor‐E2‐related factor 2; Pts, Pterostilbene; Sirt1, Sirtuin 1

## 4 DISCUSSION

Pts can not only inhibit oxidative stress and allergic inflammation, but also reduce astrocytic inflammation and neuronal oxidative damage.^22^, ^23^ In addition, Pts significantly alleviates the inflammatory response of macrophages (RAW264.7) induced by lipopolysaccharide.^24^ Here, we studied the effect of Pts on oxidative stress and inflammation by a mouse model of allergic asthmas and LPS‐stimulated 16HBE cells. The main pathology of asthma is chronic airway inflammation, which often manifested by white blood cell infiltration, especially eosinophil infiltration and the shedding of epithelial cells on the airway mucosal surface.^25^ Our results showed that Pts significantly reduced goblet cell proliferation and inflammatory cell infiltration in OVA‐induced asthmatic mice. In particular, Pts prevented the infiltration of eosinophils, neutrophils and lymphocytes. Asthma is characterized by excessive activation of Th2 cells,^26^ which leads to the production of large amounts of IgE and induces allergic airway inflammation.^27^ AHR is one of the signs of asthma, and correlates positively with asthma severity.^28^ Our results showed that IL‐5, IL‐4, and IL‐13 levels in BALF were decreased by Pts but IFN‐γ was increased by Pts. In addition, IgE (total and OVA‐specific) was reduced by Pts dose‐dependently. Therefore, Pts may suppress airway inflammation in asthma by improving the imbalance of Th1/Th2 immune response.

Studies have shown that inhibiting pro‐inflammatory mediators can significantly alleviate the development of allergic asthma.^29^, ^30^ Cytokines (IL‐6, TNF‐α, and IL‐1β) by bronchial epithelial cells promotes the development of asthma.^31^ Therefore, the anti‐asthmatic effects of Pts in 16HBE cells in vitro were evaluated. The results showed that Pts treatment could significantly inhibit NO, PGE2, iNOS and COX‐2 in 16 HBE cells stimulated by LPS. In addition, Pts also reduced the levels of LPS‐induced IL‐6, TNF‐α, and IL‐1β. In addition, after Pts treatment, the G0/G1 phase of LPS‐treated cells was prolonged, and the cell's mitotic ability was restored. The above results indicate that Pts inhibits bronchial epithelial cells from producing pro‐inflammatory mediators and cytokines.

There is also oxidative stress in bronchial asthma. SOD and CAT are antioxidant enzymes that form MDA after being inactivated by ROS and membrane phospholipids.^32^ Here, the serum SOD and CAT levels in the OVA group were obviously reduced, while the MDA was significantly increased. However, Pts treatment promoted SOD and CAT, and inhibited MDA. It is shown that during chronic asthma, ROS level significantly increases.^33^ Accordingly, here, the ROS generation in 16HBE increased after LPS treatment. However, Pts significantly inhibited the induction of ROS by LPS. Therefore, the above results indicate that Pts reduces oxidative stress, thereby inhibiting bronchial asthma.

AMPK is a protein kinase that is sensitive to metabolism and plays an important role as an energy sensor in the absence of ATP.^34^ In this study, Pts promoted the expression of p‐AMPK in 6HBE cells, indicating that Pts may activate AMPK. The Sirt1/AMPK pathway in C2C12 muscle cells triggered by hydrogen peroxide is involved in the antioxidative stress response.^35^ Sirt1 is a NAD‐dependent histone deacetylase, which enhances AMPK phosphorylation by activating AMPK and weakens the oxidative stress response.^36^ We found that after Pts treatment, Sirt1 levels and p‐AMPK levels in cells increased significantly. The above results suggest that Pts can reduce oxidative stress and inhibit bronchial asthma through activation of AMPK/Sirt1 pathway (Figure 7).

The antioxidative stress response is carried out through Nrf2/HO‐1 pathway. It is shown that HO‐1 reduces inflammation, airway oxidative stress, excessive mucus secretion, and asthma responses.^37^, ^38^ Nrf2 is stored in the cytoplasm and binds to Keap 1 in normal state.^39^ When stimulated by oxidation, Nrf2 is translocated from cytoplasm to the nucleus, which then induces HO‐1 transcription and plays an antioxidant role.^40^ In this study, we showed that Pts upregulated the nuclear transport of Nrf2 and increased HO‐1 expression. These results indicate that oxidative stress may be attenuated by Pts via activating Nrf2/HO‐1 pathway (Figure 7).

AMPK/Sirt1 and Nrf2/HO‐1 pathways are importantly involved in reducing oxidative stress and inhibiting bronchial asthma. Activation of the AMPK pathway can reduce inflammatory lung disease.^41^ Sirt1 can inhibit the oxidative stress of vascular endothelial cells.^42^ HO‐1 was known as an anti‐inflammatory agent when human bronchial epithelium stimulated by cationic peptides.^43^ By activating Nrf2, anti‐inflammatory drugs reduce allergic airway inflammation caused by OVA.^44^ Here, we found that in 16HBE cells, Pts activated signaling pathways of AMPK/Sirt1 and Nrf2/HO‐1. In addition, siAMPK significantly reduced Sirt1, Nrf2, and HO‐1; siSirt1 reduced Nrf2/HO‐1; and siNrf2 reduced HO‐1. In 16HBE cells, we observed that Pts + compound C treatment effectively blocked the activation of p‐AMPK, Sirt1, Nrf2, and HO‐1, while Pts + EX‐527 also effectively blocked the activation of Sirt1, Nrf2, and HO‐1. Pts + ML385 also effectively prevented Nrf2 and HO‐1 expression. Together, AMPK/Sirt1 is the upstream signaling pathway of Nrf2/HO‐1.

This study has some limitations. First, animal experiments with gene knockout were not performed to verify the roles of AMPK/Sirt1 and Nrf2/HO‐1 signaling pathways. Second, the anti‐inflammatory effect of Pts was demonstrated by measuring the degree of anti‐oxidation. At present, it has been reported that the antioxidant ability of the body is closely related to the mitochondria in cells.^45^ However, the involvement of mitochondria in the antioxidant effect of Pts is not investigated in this study. Further studies are warranted.

## 5 CONCLUSION

In this study, our results reveal that Pts can relieve asthma by suppressing oxidative stress through AMPK/Sirt1 and Nrf2/HO‐1 pathways. Thus, Pts might serve as an anti‐inflammation drug for asthma.

## CONFLICT OF INTERESTS

The authors declare that there are no conflict of interests.

## AUTHOR CONTRIBUTIONS

Chang Xu and Yilan Song performed the experiments, analyzed the data and wrote the manuscript. Zhiguang Wang, Jingzhi Jiang, Yihua Piao and Li Li performed the experiments. Shan Jin and Liangchang Li searched the literatures. Lianhua Zhu and Guanghai Yan conceived the idea, designed the study, collected the funds and revised the manuscript.

## ETHICS STATEMENT

This study was approved by Institutional Animal Care and Use Committee of Yanbian University.
